# Modification of Surface Bond Au Nanospheres by Chemically and Plasmonically Induced Pd Deposition

**DOI:** 10.3390/nano11010245

**Published:** 2021-01-18

**Authors:** Heike Lisa Kerstin Stephanie Stolle, Andrea Csáki, Jan Dellith, Wolfgang Fritzsche

**Affiliations:** 1 Department of Nanobiophotonics, Leibniz Institute of Photonic Technology (IPHT), Albert-Einstein-Straße 9, D-07745 Jena, Germany; lisa.stolle@leibniz-ipht.de (H.L.K.S.S.); andrea.csaki@leibniz-ipht.de (A.C.); 2 Competence Center for Micro- and Nanotechnologies, Leibniz Institute of Photonic Technology (IPHT), Albert-Einstein-Straße 9, D-07745 Jena, Germany; jan.dellith@leibniz-ipht.de

**Keywords:** metal nanoparticles, bimetallic nanoparticles, chemical metal deposition, plasmon induced metal deposition, active plasmonics, atomic force microscopy (AFM)

## Abstract

In this work we investigated methods of modifying gold nanospheres bound to a silicon surface by depositing palladium onto the surfaces of single nanoparticles. Bimetallic Au-Pd nanoparticles can thus be gained for use in catalysis or sensor technology. For Pd deposition, two methods were chosen. The first method was the reduction of palladium acetate by ascorbic acid, in which the amounts of palladium acetate and ascorbic acid were varied. In the second method we utilized light-induced metal deposition by making use of the plasmonic effect. Through this method, the surface bond nanoparticles were irradiated with light of wavelengths capable of inducing plasmon resonance. The generation of hot electrons on the particle surface then reduced the palladium acetate in the vicinity of the gold nanoparticle, resulting in palladium-covered gold nanospheres. In our studies we demonstrated the effect of both enhancement methods by monitoring the particle heights over enhancement time by atomic force microscopy (AFM), and investigated the influence of ascorbic acid/Pd acetate concentration as well as the impact of the irradiated wavelengths on the enhancement effect. It could thus be proven that both methods were valid for obtaining a deposition of Pd on the surface of the gold nanoparticles. Deposition of Pd on the gold particles using the light-assisted method could be observed, indicating the impact of the plasmonic effect and hot electron for Pd acetate reduction on the gold particle surface. In the case of the reduction method with ascorbic acid, in addition to Pd deposition on the gold nanoparticle surface, larger pure Pd particles and extended clusters were also generated. The reduction with ascorbic acid however led to a considerably thicker Pd layer of up to 54 nm in comparison to up to 11 nm for the light-induced metal deposition with light resonant to the particle absorption wavelength. Likewise, it could be demonstrated that light of non-resonant wavelengths was not capable of initiating Pd deposition, since a growth of only 1.6 nm (maximum) was observed for the Pd layer.

## 1. Introduction

Plasmonically active metal nanoparticles exhibit many interesting features for bioanalytical sensing due to the localized surface plasmon resonance (LSPR) effect they show upon irradiation with light of resonant wavelengths. Minimum changes to the particle’s environment following changes in the LSPR peak position [1,2,3] are shown through surface-enhanced Raman spectroscopy (SERS). This is ascribed to the enhancement of the electric field around the particle due to the LSPR effect of the metal nanoparticle [4,5,6]. Cancer treatment is another field of application for plasmon-active metal nanoparticles based on their capacity to transform irradiated light to heat, enabling photothermal tumor therapy in the presence of oxygen to form reactive oxygen species (ROS) and destroy tumor cells [7,8,9]. In the field of catalysis, plasmon-active metal nanoparticles play a vital role due to localized surface plasmon resonance, above all when the excitation of the particle results in the formation of highly energetic electrons which can catalyze different reactions such as reduction processes [10,11,12,13,14]. A special area of research is focused on bimetallic nanoparticles, which are available in different material combinations, e.g., AuAg [15,16,17,18], AuPd [14,19,20], AuPt [21], or AgPd [22,23]. These are utilized for SERS measurements, [6,24,25,26,27] sensors, [21,28,29], and photovoltaic [30] or catalytic applications [14,31,32,33].

Bimetallic nanoparticles can be synthesized as linked monometallic, core–shell, and alloyed structures. In the case of core–shell particles, the core particle is fully covered with a layer of the second metal, whereas in the case of alloyed structures the layer may not be complete and only certain parts of the nanoparticles are covered with the second metal, resulting in a composite of both metals. In linked monometallic structures, two particles of different metals are “merged”, forming a new bimetallic nanoparticle [34]. Other structures involve hollow bimetallic nanoparticles like those obtained after anodic dissolution processes [35,36]. The formation of such bimetallic nanoparticles is mostly carried out using wet chemical synthesis and bottom-up approaches [18,36,37,38], utilizing reducing agents such as sodium borohydride, hydrazine [37,39], or ascorbic acid [18]. Two metal salts of different metals are thus simultaneously reduced, which results in randomly linked monometallic or alloyed structures. If a more defined structure like in case of core–shell particles is desired, the successive synthesis of each layer must be carried out in order to guarantee a regular core–shell structure [36,37].

Bimetallic particles with gold as the main part and palladium as additive metal are discussed in the literature due to their promising catalytic activity [40,41,42]. Gold nanoparticles can be synthesized in different shapes and sizes, thereby covering the entire range of visible light with regard to their activation wavelength [2,43,44]. The gold part in a linked monometallic structure can thus serve as the “antenna” responsible for plasmonic resonance (and thereby activation) and light collection, whereas palladium represents the catalytic active part [40,41], e.g., for carbon–carbon coupling reactions, [45,46] CO_2_ reduction [47], or hydrogen generation [48]. Regarding alloyed and core–shell bimetallic nanoparticles, an interesting feature is their localized surface plasmon resonance (LSPR) excitation wavelength. The former shift with the alloy composition, whereas in case of the latter the outer shell plays a significant role after complete coverage [36].

Nakamura et al. [49] succeeded in obtaining monometallic nanoparticles in light-activated synthesis by laser irradiation of an aqueous HAuCl_4_ solution. This approach is also possible for bimetallic alloy particles, as shown by Herbani et al. for Au-Ag particles from HAuCl_4_/AgNO_3_ metal salts dissolved in hexane [50]. In other experiments conducted by McGilvray et al. [51], Irgacure 2959 was utilized as electron source through the photochemical generation of a ketyl radical by ultraviolet A (UVA) radiation in the presence of gold seeds and AgNO_3_ in order to obtain Au-Ag core shell bimetallic nanoparticles.

In our experiments we utilized the plasmonic effect of gold nanospheres. The light of wavelengths that were either resonant or non-resonant to the absorption wavelength of the utilized nanoparticles was applied. When irradiating the nanoparticles, different processes can occur, such as the transformation of the energy of light into heat formation, enhancement of the local field, or the generation of hot electrons [52,53]. The formation of hot electrons is of special interest for our experiments, as these can be passed on to other molecules, inducing splitting, oxidation, and reduction reactions [52,54]. Therefore, it is expected that the electrons generated on the surface of the gold nanospheres can lead to the reduction of Pd acetate there, and atomic Pd is deposited as a result. It is thus assumed that core-shell particles are formed, as extensive studies of similar systems of Au/Ag core/shell particles formed due to the electroless reduction of Ag salts in solution have clearly documented the formation of a separate coating (shell) around the original particle, as shown by high-resolution transmission electron microscopy (HR-TEM) images [29,55]. Analogous results have been reported for other metal combinations, such as the Au/Pd core/shell systems investigated here [56,57].

In order to classify our experiments, in Table 1 a number of similar synthesis approaches of bimetallic nanoparticles obtained from light-driven photochemical techniques as well as from experiments with reducing agents are listed for comparison.

In this paper we demonstrate the Pd enhancement of gold nanospheres bound to a silicon surface by a classical wet chemical reduction using ascorbic acid. We thereby wish to observe the effect of the ascorbic acid and Pd acetate concentration, respectively. In other experiments we exploit the plasmonic effect as a method to reduce Pd acetate without further reduction agents and aim to prove this effect by control experiments with light of non-resonant wavelengths.

## 2. Materials and Methods

### 2.1. Chemicals and Materials

All utilized chemicals were obtained commercially and used without further purification: HAuCl_4_-trihydrate (99.5% Au content), citric acid, ascorbic acid (AS), 3-aminopropyl triethoxy silane (APTES), and the solvents acetone, ethanol, and ROTISOL^®^ were obtained from Carl Roth GmbH & Co KG (Karlsruhe, Germany). Pd(AcO_2_)_2_ (PdAc, 47% Pd content) was purchased from Merck KGaA (Darmstadt, Germany). The 80-nm gold nanospheres were obtained from BBI Solutions (Crumlin, UK). For solution preparation, solely Milli-Q water was used.

### 2.2. Synthesis of 30-nm Gold Nanospheres

For this study, 30-nm gold nanospheres were synthesized following the prescription by Bastus et al [63]. First, gold seeds were obtained by preparing a sodium citrate solution (2.2 mM, 150 mL), which was heated for 15 min under vigorous stirring. After boiling, HAuCl_4_ solution (25 mM, 1 mL) was added and the mixture left for 10 min. When the solution was cooled down to 90 °C, HAuCl_4_ solution (25 mM, 1 mL) was added and the solution was left stirring for 30 min. The addition of HAuCl_4_ solution was then repeated and after 30 min, 55 mL of the obtained nanoparticle solution was removed and Milli-Q water (53 mL) as well as sodium citrate solution (2.2 mM, 2 mL) were added. This process of HAuCl_4_/sodium citrate addition and removal of nanoparticle solution was repeated until a precipitation of nanoparticles was observed. Each 55-mL sample of removed gold nanosphere solution provided a certain particle size diameter. For the Pd enhancement experiments, the first fraction removed was utilized.

### 2.3. Characterization of Gold Nanosphere Solutions

Both solutions with gold nanospheres (AuNPs) were characterized by ultraviolet-visible (UV/VIS) spectroscopy utilizing a Thermo Fisher NanoDrop One^C^ (Waltham, MA, USA) and a JASCO V-670 UV-VIS-NIR (Easton, PA, USA) spectrophotometer as well as scanning electron microscopy (SEM) imaging (Figure S1, JEOL FE-SEM JSM 6300F and JSM-7900F, Akishima, Japan).

### 2.4. Binding of Gold Nanospheres on Silicon Nickel/Chromium Grid Chips

Silicon chips (7.5 mm × 7.5 mm) with a 25 × 25 cross-hatch pattern of nickel/chromium line grid dividing the chip into different fields from A01 to Y25 were prepared using a standard photolithographic process. To remove their protective layer, a removal solution consisting of an alkaline solution on an ammoniac base was used prior to a wash with distilled water and then a short wash in ethanol. The chips were then cleaned using acetone, ROTISOL^®^, ethanol, and Milli-Q water in an ultrasonic bath for 10 min for each step. Finally, they were dried under nitrogen flow and activated by oxygen plasma (*t* = 30 min, *p* = 380 W and *p* = 1.6 mbar), utilizing a 200-G Plasma System (Technics Plasma GmbH, Kirchheim bei München, Germany) with a Leybold Trivac pump (Köln, Germany).

Within 10 min after activation a layer of APTES was applied to the chips, following the slightly modified method of Fang and Hoh [64]. First, an APTES solution was prepared from 100-mL ultrapure water, which was stirred slowly and to which 1 mL of acetic acid (0.1 M) and 1 mL of APTES were added. The hydrolyzation of APTES was carried out at least for 10 min before use. Then, the APTES solution was poured over the activated chrome grid chips and left on an orbital shaker for 1 h at 150 rpm. In the final step, the chips were removed from the solution, the surplus APTES washed off with Milli-Q water, and the chips dried under nitrogen flow and stored under argon.

After 24 h, the chips were again cleaned in the ultrasonic bath in ultrapure water shortly before the respective particle solution was diluted with Milli-Q water (particles: Milli-Q water = BBI Solutions 80-nm gold nanospheres 1:5, Bastus nanoparticles 1:10, corresponding to 1.18 × 10^11^ P/mL and 2.34 × 10^12^ P/mL, respectively, as obtained from UV/Vis data following the method by M.A.Hayat [65]). This was poured over the chips so that all chips were completely covered by liquid and left in an orbital shaker at 150 rpm for 1 h. The chips were then washed again with Milli-Q water and dried under nitrogen flow.

### 2.5. Metal Enhancement with Palladium Acetate and Ascorbic Acid

The palladium acetate and ascorbic acid solutions were added to each chip in varying concentrations, whereby the volume ratio *Ψ* [mL] of both solutions was overall 1:1, utilizing 4 mL of each solution. There were two variations. Firstly, the palladium acetate (PdAc) concentration was varied at 1.0 mM, 1.5 mM, and 2.5 mM, while the ascorbic acid (AS) concentration remained constant at 228 mM. In the second variation, the palladium acetate concentration was set to 2.5 mM and the ascorbic acid concentration varied at 57 mM, 114 mM, and 228 mM. New solution was added every two minutes. During the enhancement processes the chips in solution were continuously shaken on an orbital shaker at 150 rpm.

### 2.6. Light-Activated Metal Enhancement with Palladium Acetate

In the case of the plasmon-induced metal enhancement, the chip coated with AuNPs was placed in a petri dish, and palladium acetate solution (2.5 mM) was added in a volume that was sufficient to cover the chip completely. Then, a halogen 150-W lamp emitting white light (Dolan-Jenner Fiber-Lite^®^ Mi-150, Boxborough, MA, USA) at maximum power was placed over the chip at a distance of 10 cm and the light was switched on. The reaction was carried out in 2-min steps up to 10 min, and then for 10 min straight without intermediate steps. In further tests, three quadratic arrays were utilized emitting green, blue, or red light, each consisting of 64 light-emitting diodes (LEDs) obtained from Super Bright LEDs Inc. (St. Louis, MO, USA). The green LED array (λ = 516 nm measured, manufacturer’s specification: 523 nm) was used with the 30-nm gold spheres which were resonant at the given wavelength. For control experiments, 80-nm gold nanospheres (resonant at 557 nm) were utilized and irradiated with the green, blue (457 nm measured, manufacturer’s specification: 470 nm), and red (628 nm measured, manufacturer’s specification: 625 nm) LED array. The LED arrays were installed 5 cm above the chips placed in Pd acetate solution. The intensity of the Dolan-Jenner lamp was measured at a distance of 10 cm using an L30A-SH-V1 detector (*Ø* = 2.8 cm) with a Nova II laser power energy meter (Ophir Optronics LTD, Jerusalem, Israel), and was estimated to be 12.3 mW∙cm^−2^. The LEDs were measured at a distance of 5 cm from the detector, yielding an intensity of 6.17 mW∙cm^−2^ in the case of the green LEDs, 10.4 mW∙cm^−2^ for the red LEDs, and 30.0 mW∙cm^−2^ for the blue LEDs. The lamp spectra were verified by measurements with a Flame UV-VIS Miniature Spectrometer (Ocean Optics Inc., Largo, FL, USA) with an exposure time of 15 ms and a distance of 73.5 cm to the fiber. Temperature measurements were carried out in extra experiments with chips identical to those used in the former experiments. The Pd acetate solution and the chip temperature before and directly after 2 and 10 min of irradiation were monitored with a RS 1327 infrared thermometer (RS Components GmbH, Frankfurt/M., Germany).

### 2.7. Characterization of the Immobilized Nanoparticles before and after Metal Enhancement by Atomic Force Microscopy (AFM), Scanning Electron Microscopy (SEM), and Scanning Electron Microscopy Coupled with Energy-Dispersive X-ray Spectroscopy (SEM-EDX)

The height increase of the particles due to metal enhancement was analyzed by AFM measurements, utilizing a NanoScope V, Dimension Icon with ScanAsyst system (Bruker Corporation, Billerica, MA, USA) and a NanoScope 3D, Dimension^TM^ 3100 system (Veeco Metrology Group Digital Instruments, Plainview, NY, USA). Therefore, each chip was measured before the metal enhancement experiments and after each enhancement step. For each chip, an area of 5 µm (15 µm in case of very sparse particle coating) was scanned with 512 samples per line. In order to relocate the measurement position after the enhancement steps again, the same field of each chip was chosen for measurement, as indicated by a lithographic crosshatch chromium pattern marked by letters and numbers and with the help of the internal coordinates of the NanoScope 6.14R1 (Veeco Metrology Group Digital Instruments, Plainview, NY, USA) and NanoScope 9.0 software (Bruker Corporation, Billerica, MA, USA). For calculations, the heights of particles of the measured field were determined by the software Gwyddion 5.1 (David Nečas and Petr Klapetek, Czech Metrology Institute, Brno, CZ) and were averaged out.

SEM imaging of the chips was carried out with a SEM system JEOL JSM-7900F (Akishima, Japan). Measurement conditions are shown in Figure 3: acceleration voltage (Vacc) = 15.0 kV, detector = upper electron detector (UED), magnification = 50,000 (Figure 3a) and 150,000 (Figure 3b); Figure 8: Vacc = 15.0 kV, detector = UED, magnification = 30,000 (Figure 8a) and 50,000 (Figure 8b); Figure S1: Vacc = 15.0 kV, detector: UED, magnification 100,000 (Figure S1a) and 50,000 (Figure S1b); and Figure S2: Vacc = 15.0 kV, detector: UED, magnification 25,000. For SEM-EDX analyses, an FE-SEM system JSM-6700F from JEOL (Akishima, Japan) and an ED spectrometer with silicon drift detector XFlash 5030 from Bruker Nano GmbH (Berlin, Germany) were used. Measurement conditions are shown in Figure 11: Vacc = 10.0 kV, detector = secondary electron images (SEI), magnification = 80,000; and Figure 12: Vacc = 15.0 kV, detector = SEI, magnification = 100,000. The SEM-EDX samples were coated with approximately 5 nm of carbon prior to measurements.

## 3. Results

### 3.1. Pd Enhancement of 80-nm AuNPs by Reduction of Pd Acetate with Ascorbic Acid

**Figure 1:**
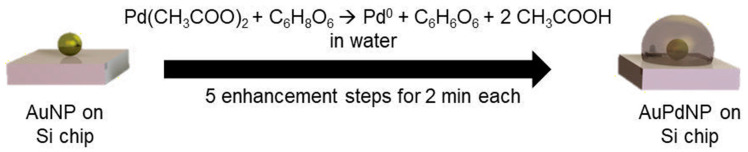
Formation mechanism of AuPd nanoparticles (AuPdNP) from Au nanoparticles (AuNP) by reduction of Pd acetate with ascorbic acid to Pd^0^, dehydroascorbic acid, and acetic acid.

In a first set of experiments, the effect of reducing agent concentration was studied. Variations were therefore made regarding the concentration of ascorbic acid (57, 114, and 228 mM), with a stable Pd acetate concentration of 2.5 mM. In a second experiment, the Pd acetate concentration was varied at 1.0, 1.5, and 2.5 mM, with a stable concentration of ascorbic acid of 228 mM in order to monitor changes when less Pd acetate was available for reduction to Pd^0^. The formation mechanism of possible AuPd nanoparticles (AuPdNPs) was based on the reduction of Pd acetate by ascorbic acid as a reducing agent described in Figure 1.

The palladium acetate was reduced in the presence of the immobilized gold nanoparticles to Pd^0^ and acetic acid by the addition of ascorbic acid, which was oxidized to dehydroascorbic acid. Through the addition of fresh Pd acetate/ascorbic acid solutions in five steps at two minutes each, a constant deposition of Pd on the AuNPs took place, leading to a consistent growth of the Pd layer around the AuNP.

**Figure 2:**
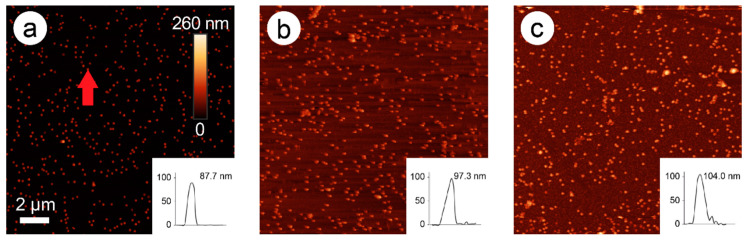
Atomic force microscopy (AFM) images of 80-nm gold nanospheres (AuNPs) after 0 min (**a**), 4 min (**b**), and 10 min (**c**) of enhancement with 1.5 mM palladium acetate (PdAc) and 228 mM ascorbic acid. Insets: Height profiles of the marked nanoparticle (**a**, red arrow) over time. The scale bar of image 1a pertains to all three images.

In Figure 2, an AFM image time series of the development of particle height over time is presented (here: 228 mM ascorbic acid and 1.5 mM Pd acetate).

In these images, an exemplary AuNP was chosen, and its height followed over time (0, 4, and 10 min). The original bare gold nanoparticle exhibited a height of 87.7 nm, which was increased to 97.3 nm after two steps of enhancement (4 min), and to 104 nm after five steps of enhancement (10 min). New particles were also formed over time, probably representing pure Pd nanoparticles.

**Figure 3:**
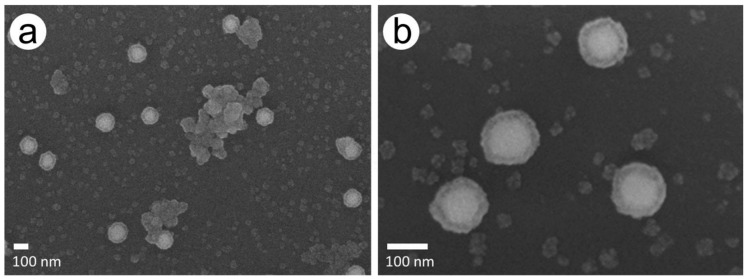
SEM images of the chip with 228 mM AS and 1.5 mM PdAc after a total enhancement time of 10 min. (**a**) Agglomerates and newly formed Pd particles besides AuPd particles. (**b**) Close-up of AuPd particles.

This effect was confirmed by SEM imaging, which showed smaller Pd particles of different sizes and partially larger agglomerates besides AuPdNPs, which at times formed clusters during the Pd enhancement process (Figure 3a). A close-up of the Pd-covered particles revealed spherical particles apparently covered with a complete Pd shell with a quite homogeneous thickness of around 20 nm (Figure 3b).

In the other enhancement experiments, smaller, newly formed particles could be observed (Supporting Materials, Figure S2). When comparing the development of particle height of the Pd-enhanced AuNPs over time (see Figure 3; for better visibility displayed without error bars and further information see Figure S3), it can be seen that the concentration of ascorbic acid indeed makes an impact on the height of the formed palladium shell on the gold spheres, but the palladium acetate concentration also plays a role.

**Figure 4:**
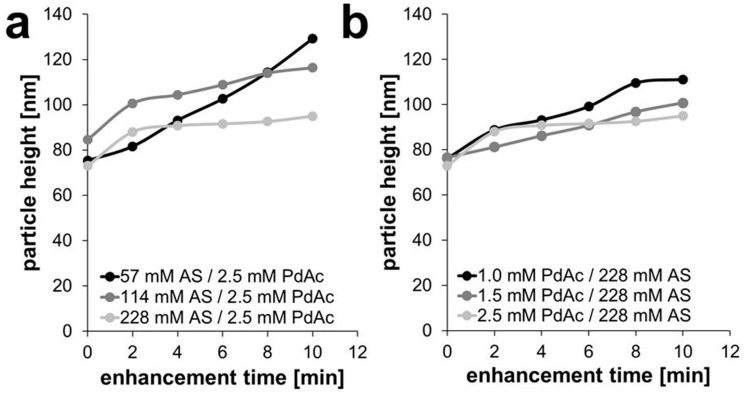
Development of particle weight during experiments with (**a**) a constant Pd acetate concentration of 2.5 mM and (**b**) a constant ascorbic acid concentration of 228 mM.

When varying the reducing agent ascorbic acid at 57, 114, and 228 mM (for a constant Pd acetate concentration of 2.5 mM), an initial and almost linear growth behavior was observed for the lowest ascorbic acid concentration, with a growth rate of 3.15–5.40 nm/s; this behavior slowed down for higher AS concentrations, with values of 8.0–3.18 nm/min and 7.50–2.20 nm/min, respectively (Figure 4a and Table S1). For 228 mM AS, only the first minutes showed significant growth; later, the height stayed rather unchanged with a plateau.

When following the reactions at constant concentration of reducing agent (228 mM AS) for varying Pd acetate concentrations of 1.0, 1.5, and 2.5 mM (Figure 4b and Figure S3), again the growth rate decreased with increasing concentrations of PdAc. There was an almost linear growth behavior at the lowest concentration of 1.0 mM with a growth rate of 6.30–3.50 nm/min, and a perfect linear growth for the concentration of 1.5 mM PdAc with a growth rate of 2.30–2.42 nm/min. Then, a flattening of the curve of the experiment occurred at 2.5 mM PdAc, with a growth rate of 7.50–2.20 nm/min (Figure 4b and Table S1).

When analyzing the thickness growth of the Pd shell over 10 min of enhancement and comparing all five experiments, it could be observed that the total height of Pd shell was the lowest in case of 2.5 mM PdAc and 228 mM AS (22 nm) and the highest for 2.5 mM PdAc and 57 mM AS (54 nm). An average palladium shell thickness of 31 nm was determined for 114 mM AS, of 24 nm for 228 mM AS with 1.5 mM PdAc, and 35 nm for 228 mM AS and 1.0 mM PdAc.

The reason for this reaction behavior can be seen in both test series in the tendency to form new particles solely consisting of palladium induced by new primary nucleation, instead of enhancing the gold nanoparticles as seen in the AFM (Figure 2) and SEM images (Figure 3). This effect is especially visible for the highest ascorbic acid concentration of 228 mM with 2.5 mM PdAc, as this reaction curve flattened after the first 2-min step of Pd enhancement. Although the same amount of Pd acetate and ascorbic acid was added in every step, the tendency was to form rather new Pd particles instead of further enhancing the already covered AuNPs, especially in case of the highest concentration of ascorbic acid (228 mM) and the highest concentration of PdAc (2.5 mM). The shell growth almost stagnated. In the other enhancement experiments, the effect of the formation of new particles could also be observed, although without Pd shell growth to the same extent as for the highest ascorbic acid concentration. For the experiments with a constantly high ascorbic acid concentration of 228 mM and varying Pd acetate concentrations of 1.0, 1.5, and 2.5 mM, pure Pd particles could be observed on the surface, with partial linking of AuNPs to form clusters. Here, it could be seen that the higher the Pd acetate concentration, the lower the total height of the Pd shell.

### 3.2. Light-Induced Pd Enhancement of 80-nm and 30-nm AuNPs

**Figure 5:**
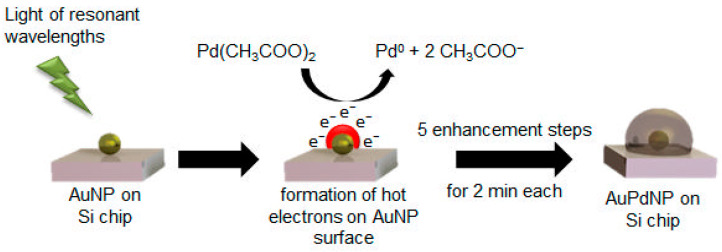
Formation mechanism of AuPd nanoparticles (AuPdNP) by light-induced reduction of Pd acetate by hot electrons generated through the irradiation of the Au nanoparticles (AuNP) with light of resonant wavelengths.

The expected mechanism for the formation of AuPd nanoparticles through light-induced Pd enhancement is displayed in Figure 5. The light of wavelengths resonant to the absorption wavelength of the utilized nanoparticle sample was necessary in order to initiate localized surface plasmon resonance. Through this process, hot electrons could be formed on the particle surface, which were then capable of reducing the added Pd acetate in aqueous solution to Pd^0^. The atomic Pd was deposited on the AuNP surface in each of the five steps for two minutes, successively enhancing the AuNPs with Pd and leading to a bimetallic nanoparticle.

**Figure 6:**
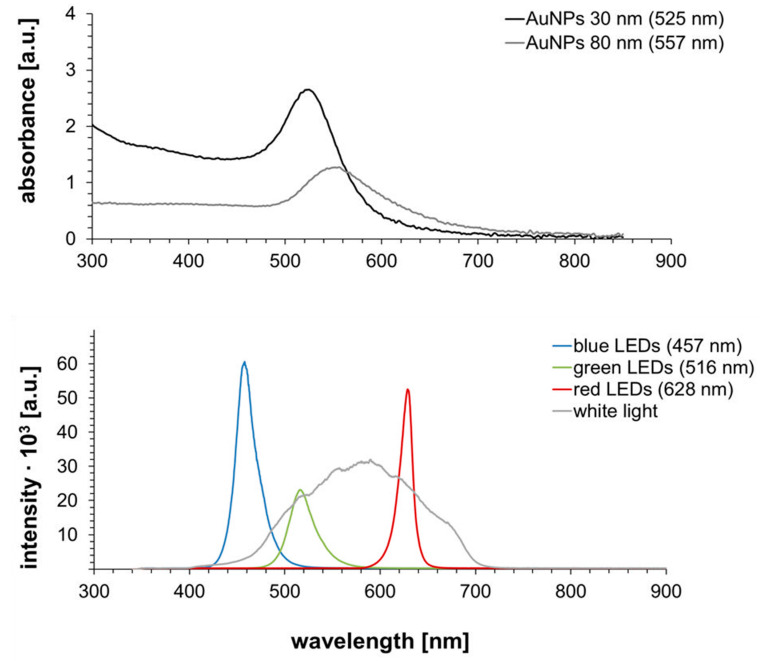
UV/Vis spectra of 30 and 80 nm Au NPs (**top**) and spectra of utilized light sources (**bottom**).

For initial tests of the effect of light on Pd enhancement of gold nanospheres, 80-nm particles were irradiated by white light. In order to determine the influence of light resonant on the plasmonic resonance of particles, monochromatic light sources (LED arrays) emitting wavelengths both at as well as away from the resonant wavelength were used for the irradiation of 30-nm gold nanoparticles (resonant at 525 nm) and later in control experiments with 80-nm AuNPs (resonant at 557 nm). The UV/Vis spectra of both AuNPs samples in the utilized dilution as well as the spectra of the used light sources are displayed in Figure 6.

The UV/Vis spectra revealed a clear difference in absorption wavelength due to the different particle sizes. Due to their smaller size, the 30-nm AuNPs showed a lower wavelength than the 80-nm AuNPs. Furthermore, it could be seen that the optical density of these particles was higher in the chosen dilution than for the 80-nm AuNPs, as this facilitated the retrieval of the smaller particles and particle patterns after enhancement in the AFM measurements. When analyzing the spectra of the light sources, the ranges of wavelengths covered by the LED arrays was expectedly narrower than the spectra of the lamp emitting white light, which covered a range of around 400–700 nm. Therefore, green LEDs and white light close to or at the absorption wavelengths of the AuNPs were expected to show a significant enhancement effect in comparison to the blue and red LED arrays.

**Figure 7:**
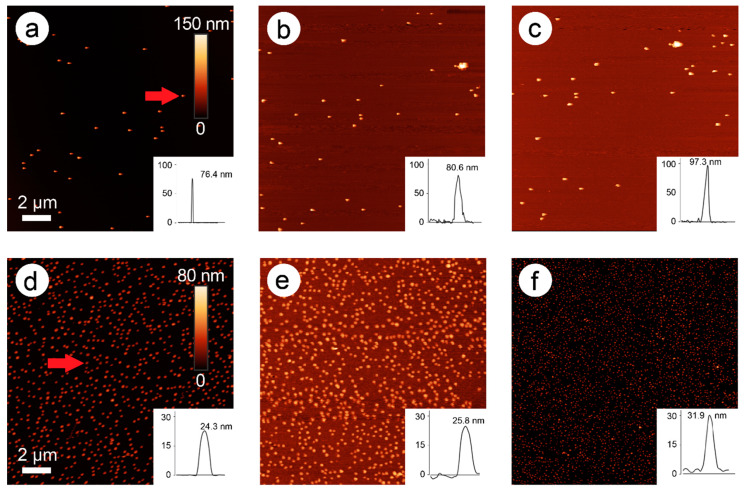
(**a**–**c**) AFM images of Pd enhancement of 80-nm AuNPs with white light and (**d**–**f**) of 30-nm AuNPs with green light with captures of 0, 4, and 10 min, respectively. Insets: Height profiles of the marked particle (red arrow) over time. The scale bar of image 6a also pertains to images b and c and the scale bar of image 6d also pertains to images e and f.

The reaction was followed by AFM measurements in two-minute steps. Figure 7 presents selected AFM images (including particle height measurements) of the enhancement of 80-nm AuNPs with white light (Figure 7a–c) and of 30-nm AuNPs with green LEDs (Figure 7d–f).

**Figure 8:**
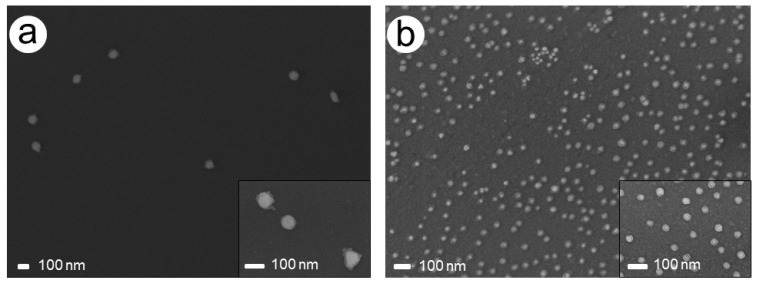
SEM images after irradiation of AuNPs in PdAc solution: (**a**) 80-nm AuNPs (white light) and (**b**) 30-nm AuNPs (green light). Insets show a close up of the respective particles.

When comparing the images and the height profiles over time, a Pd enhancement of both samples becomes apparent. Compared to the experiments with the classical chemical reduction with ascorbic acid, the thickness of the Pd shell and therefore the total particle height was lower. Interestingly, no additional, pure Pd particles were seen, unlike in the experiments with ascorbic acid. This is a good indicator that indeed reduction of Pd acetate in a close environment to the gold nanoparticles took place, which can be explained by the release of hot electrons from the particle surface to Pd acetate located in immediate vicinity, leading exclusively to enhancement of these particles. In this case, SEM images were carried out and revealed that the Pd layer was not as homogeneous as for the experiments with ascorbic acid, especially in case of white light, but was in both cases well visible (Figure 8).

**Figure 9:**
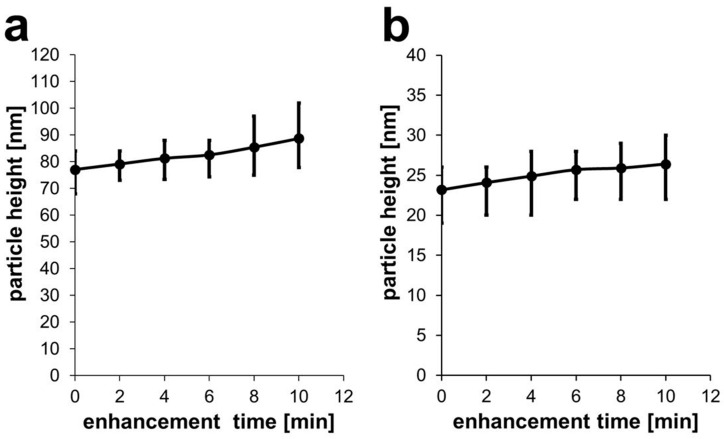
(**a**) Development of particle height after each enhancement step of two minutes for 80-nm AuNPs and white light and (**b**) for 30-nm AuNPs with green light.

White light is able to induce Pd deposition. After two minutes of irradiation of 80-nm AuNPs, a Pd layer of about 2 nm was formed on the particle surface. Additional two-minute steps resulted in total heights of 4.2 nm, 5.5 nm, 8.38 nm, and 11.65 nm, respectively (Figure 9a).

Utilizing monochromatic light of the resonance wavelength to irradiate 30-nm AuNPs, a 2-min enhancement resulted in a layer thickness of about 0.9 nm. Subsequent steps led to 1.7 nm, 2.5 nm, 2.7 nm, and 3.2 nm thicknesses, respectively (Figure 9b).

For white light irradiation of 80-nm AuNPs, the growth of the Pd shell was almost linear, with a continuous growth of around 1 nm/min (compare Table S2, Supporting Materials). Compared to this, for green light irradiation of 30-nm AuNPs, the Pd shell growth reached a plateau, with the growth rate decreasing from 0.45 nm/min to 0.32 nm/min over time (Table S2, Supporting Materials). This could be due to a shift in the plasmonic resonance due to Pd deposition: The resonance of the generated Au/Pd core/shell particle is different from that of a homogeneous Au nanoparticle, so that the green light is not exciting at exact resonance, resulting in a lower efficiency.

The particle size of the starting particles themselves might have an impact on the enhancement process. In this regard, Festag et al. reported an influence of particle size in Ag and Au enhancement experiments of gold nanospheres and demonstrated that smaller particles showed less increase in particle height over time than larger ones [59]. Therefore, experiments clarifying the influence of particle size and the different light sources and their wavelengths were carried out.

### 3.3. Influence of the Wavelength of the Irradiated Light

**Figure 10:**
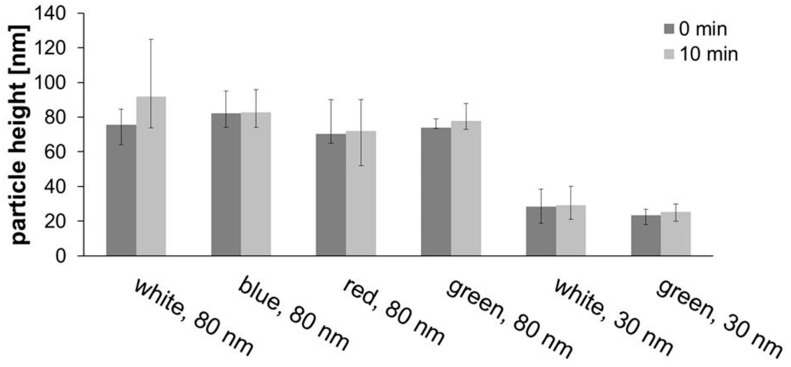
Development of particle height in experiments with different light sources with 80 and 30 nm AuNPs for 10 min.

Further tests were carried out both with white light but also with blue, red, and green LEDs with 80-nm AuNPs for 10 minutes, and with white and green light for 30-nm AuNPs. For the 30-nm particles, a resonance wavelength of 525 nm (Figure 6) was observed, so that the green light would have the greatest impact, but not the other colors. The effect of nanoparticle size was investigated by comparing the results of 10-min white or green irradiation of 80-nm with 30-nm particles (Figure 10 and AFM images in Supporting Materials Figure S4).

When irradiating 80-nm particles with white light, a significant increase in height was observed, especially for 10 min of straight irradiation (16.4 nm), but also for five periods of 2 min each (9.8 nm). Blue light led to no observable effect, with red only a slight increase (1.6 nm) was observed, but for green a significant growth of 4.1 nm was found. Apparently, spectrally selected light of or near resonance wavelengths show some effects, but not when far away. For the effect of particle size, when white light was used, 80-nm particles grew by 16.4 nm, compared with only 0.8 nm for 30-nm particles. The same applied to green light with 2.1 nm (for 30-nm AuNPs) and 4.1 nm (for 80-nm AuNPs).

Furthermore, temperature effects were evaluated, since besides electrons provided by reducing agents or (as hot electrons) by plasmonic effects on metal nanostructures, temperature effects can also result in the reduction of PdAc, as it will decompose above 200 °C [66]. Because increases in temperature of plasmonic nanostructures under resonant light irradiation (plasmonic heating) have been reported [67], this effect was investigated for the studied system (Supporting Materials, Table S3). However, no significant effect could be found and it can be concluded that (plasmonic) heating does not play a significant role in the light-induced Pd enhancement process.

### 3.4. Evaluation of Structure of the Formed Bimetallic Nanoparticles

For closer evaluation of the structure of the obtained bimetallic nanoparticles, energy-dispersive X-ray spectrometry in combination with scanning electron microscopy (SEM-EDX, EDX spectra in the Supplementary Materials, Figures S6 and S7) was carried out for an exemplary sample using the classical reduction with ascorbic acid (chip with 80-nm AuNPs and 114-mM AS, 2.5-mM PdAc) as well as plasmonic enhancement (chip with 80-nm AuNPs and white light). The SEM images shown in Figure 3 and Figure 8 lead to the suggestion that the particles exhibit a core–shell structure with the AuNPs as core particles and a complete Pd shell. This can also be derived from the two enhancement techniques, in which a core gold particle is always coated with palladium step-by-step.

**Figure 11:**
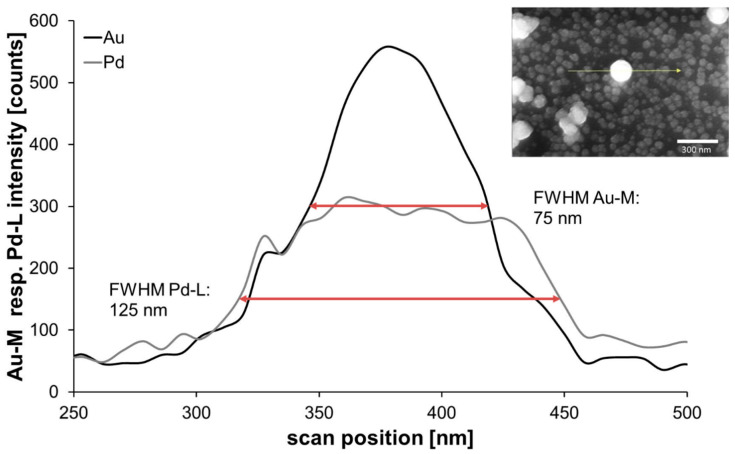
Energy-dispersive X-ray spectrometry (EDX) line analysis of an exemplary AuPd nanoparticle (inset, chip with 80-nm AuNPs and 114-mM AS, 2.5-mM PdAc).

Beginning with the chip from classical reduction, with help of an EDX line-scan, it was possible to observe the positions of the two metals in a chosen particle (Figure 11). Through comparing the shape of signals and the full width at half maximum (FWHM), it can be stated that the signal of gold can be found in the core region with a quite narrow FWHM of 75 nm. In contrast, the line-profile of the encasing Pd was expectedly broader, with a FWHM of 125 nm, indicating a complete covering of the accessible surface of the immobilized gold nanoparticle as outlined in Figure 1 and Figure 5, which showed a more even distribution along the measured line, indicating a complete covering of the gold nanoparticle.

**Figure 12:**
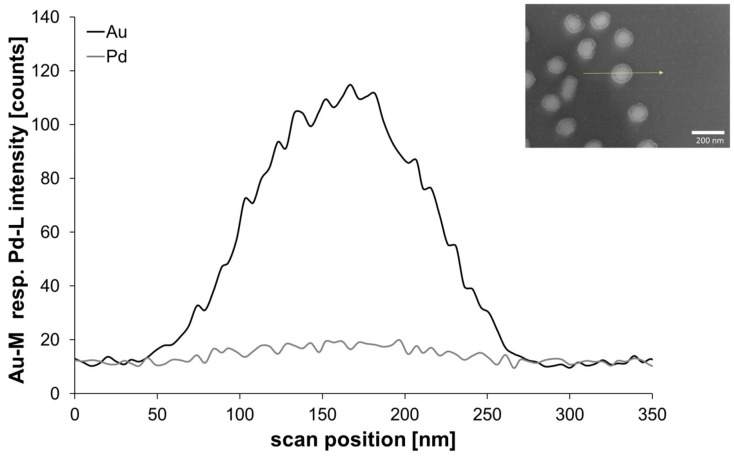
EDX line analysis of an exemplary AuPd nanoparticle (inset, chip with 80-nm AuNPs and 2.5-mM PdAc and step-wise irradiation with white light).

The EDX line-scan from an exemplary AuPd nanoparticle obtained by step-wise irradiation with white light (plasmon-induced reduction, Figure 12) revealed that palladium was in this case located around the gold core. However, the Pd layer seemed to be very thin, as the Pd signal was very low compared to the result from the reduction technique with ascorbic acid. This is not surprising when considering that only the amount of Pd acetate in the immediate vicinity of the particle surface can be reduced by the hot electrons formed during plasmon excitation.

## 4. Conclusions

In this work, we were able to demonstrate the palladium enhancement of gold nanospheres by light-induced shell growth and by a classical reduction process with ascorbic acid as a reduction agent. In comparison to the light-induced Pd enhancement, the Pd shell showed greater growth in the wet chemical enhancement experiments, but more clusters and extra (pure Pd) particles were formed, especially in case of high concentrations of reducing agent. In comparison, in the light-driven enhancement experiments a deposition of Pd seemed to be localized at the surface of the AuNPs. This leads us to the conclusion that Pd acetate was indeed reduced by hot electrons at the gold particle surface, which were formed through the plasmonic effect upon irradiation with light resonant to the absorption wavelengths of the particles. In these experiments, it was also proven that the wavelength of light utilized played a crucial role within the enhancement process. Therefore, we were able to demonstrate that with white light and green light (at which the chosen gold nanospheres are resonant) Pd shell growth could be observed, whereas with blue and red light (which are not resonant to the nanoparticles), no growth of Pd shell occurred. In these tests, an influence of the size of the starting particles could also be observed, as in tests with white and green light for enhancement of 10 minutes the 30-nm AuNPs showed overall less shell growth than the 80-nm AuNPs. Furthermore, it could be demonstrated that the heating effect of the light sources was negligible and did play any significant role in the enhancement process, which was therefore be shown to be solely based on the reduction of Pd acetate through hot electrons generated in the plasmonic excitation process. Finally, SEM images and SEM-EDX analyses suggested that the gained AuPd nanoparticles exhibited a core–shell structure with a thick Pd layer in the case of the Pd acetate reduction with ascorbic acid and a quite thin layer for light-induced Pd enhancement.

## Figures and Tables

**Table 1:** Overview of synthesis methods for different bimetallic nanoparticles and the resulting structures from light-driven techniques and experiments utilizing reducing agents.

Bimetallic System	Synthesis Method	Resulting Structure	Literature Source
**Au/Ag**	High-intensity laser irradiation of HAuCl_4_ and AgNO_3_ in hexane	Au-Ag alloys	Herbani et al. [50]
**Au/Ag**	Photochemical enhancement of Au seed solution with Ag with UVA-treated Irgacure 2959 as a reducing agent	Au/Ag core–shell nanoparticles	McGilvray et al. [51]
**Au/Ag**	Photochemical synthesis from NaAuCl_4_ and AgNO_3_ solutions with acetone/isopropanol irradiated with UV light as reducing agent in presence of CTAB (cetyltrimethylammonium bromide)	Au-Ag alloys, Au/Ag core–shell particles with pre-prepared Au seeds	Gonzalez et al. [58]
**Au/Ag**	Enhancement of surface-immobilized Au nanoparticles with Ag by reduction of Ag salts with hydroquinone	Au/Ag core–shell nanoparticles	Festag et al. [59]/Steinbrück et al. [15]
**Au/Ag**	Layering of pre-prepared Au seeds with Ag through reduction with ascorbic acid in presence of CTAB and heated NaOH in a microfluidic system	Au/Ag core–shell nanoparticles	Knauer et al. [55]
**Au/Pd**	UV irradiation of HAuCl_4_ and PdCl_2_ in solution with PEG-400 (polyethylene glycol-400) and acetone as reducing agents	Au/Pd core–shell nanoparticles	Dong et al. [60]
**Au/Pd**	Reduction of HAuCl_4_ and K_2_PdCl_6_ with ascorbic acid in solution with AgNO_3_ present	Au-Pd alloys (nanoflowers)	Ma et al. [61]
**Au/Pt**	Reduction of HAuCl_4_ by ascorbic acid/sucrose solution and formation of bimetallic nanoparticles after H_2_PtCl_6_ H_2_O addition	Au-Pt alloys	Britto Hurtado et al. [62]

## Data Availability

Raw data utilized within this article were generated at Leibniz-Institute of Photonic Technology, Jena, Germany. The data presented in this study are available on request from the corresponding author (W.F.).

## Supplementary Materials

The following are available online at https://www.mdpi.com/2079-4991/11/1/245/s1, Figure S1: SEM images of utilized gold nanospheres (a) 30 nm and (b) 80 nm; Figure S2: AFM images (a1–d1) and SEM images (a2–d2) of 80-nm AuNPs after 10 minutes of enhancement in 2-min steps. Images (a): 57-mM AS and 2.5-mM Pd acetate, (b): 114-mM AS and 2.5-mM PdAc, (c): 228-mM AS and 2.5-Pd acetate, (d): 228-mM and 1.0-mM Pd acetate. The scale bar of image S 2 a1 pertains to all four images; Figure S3: Effect of reducing agent concentration on particle height development at constant (2.5 mM) Pd acetate concentration (a–c), and at constant ascorbic acid concentration of 228 mM (d–f); Table S1: Total particle height, height of Pd shell and growth kinetics (nm/min) of 2-min enhancement steps up to 10 min for Pd enhancement with ascorbic acid; Table S2: Total particle height, height of the Pd shell, and growth kinetics (nm/min) of 2-min enhancement steps up to 10 min of 80-nm AuNPs under white light and of 30-nm AuNPs under green light; Figure S4: AFM images of Pd enhancement with light at 0 and 10 min (without 2-min steps) and reaction times (a1–2: 80-nm AuNPs/white light, b1–2: 80-nm AuNPs/blue LEDs, c1–2: 80-nm AuNPs/red LEDs, d1–2: 80-nm AuNPs/green LEDs, e1–2: 30-nm AuNPs/green LEDs, f1–2: 30-nm AuNPs/white light). The scale bar displayed in the respective first image is analogous within each set of images; Table S3: Temperature values [°C] and differences [K] of the chrome grid chip and the Pd acetate solution before and after irradiation during experiments; Figure S5: UV/Vis spectrum of Pd acetate solution (2.5 mM); Figure S6: EDX spectrum of a nanoparticle cluster on the chip with 80 nm AuNPs and 114 mM AS / 2.5 mM PdAc (carbon signal due to carbon coating of the sample, oxygen signal due to oxidized silicon and silicon signal itself from the chip); Figure S7: EDX spectrum of a chosen nanoparticle on the chip with 80 nm AuNPs and 2.5 mM PdAc and step-wise irradiation with white light (carbon signal due to carbon coating of the sample, oxygen signal due to oxidized silicon and silicon signal itself from the chip).

## References

[1] Mayer K.M., Hafner J.H. (2011). Localized Surface Plasmon Resonance Sensors. Chem. Rev..

[2] Csáki A., Thiele M., Jatschka J., Dathe A., Zopf D., Stranik O., Fritzsche W. (2015). Plasmonic nanoparticle synthesis and bioconjugation for bioanalytical sensing. Eng. Life Sci..

[3] Pittner A., Wendt S., Zopf D., Dathe A., Grosse N., Csáki A., Fritzsche W., Stranik O. (2019). Fabrication of micro-patterned substrates for plasmonic sensing by piezo-dispensing of colloidal nanoparticles. Anal. Bioanal. Chem..

[4] Chen H.-Y., Lin M.-H., Wang C.-Y., Chang Y.-M., Gwo S. (2015). Large-Scale Hot Spot Engineering for Quantitative SERS at the Single-Molecule Scale. J. Am. Chem. Soc..

[5] Caridad J.M., Winters S., McCloskey D., Duesberg G.S., Donegan J.F., Krstić V. (2017). Hot-Volumes as Uniform and Reproducible SERS-Detection Enhancers in Weakly-Coupled Metallic Nanohelices. Sci. Rep..

[6] Žukovskaja O., Agafilushkina S., Sivakov V., Weber K., Cialla-May D., Osminkina L., Popp J. (2019). Rapid detection of the bacterial biomarker pyocyanin in artificial sputum using a SERS-active silicon nanowire matrix covered by bimetallic noble metal nanoparticles. Talanta.

[7] Rejiya C.S., Kumar J., Raji V., Abraham A. (2012). Laser immunotherapy with gold nanorods causes selective killing of tumour cells. Pharmacol. Res..

[8] Sztandera K., Gorzkiewicz M., Klajnert-Maculewicz B. (2019). Gold Nanoparticles in Cancer Treatment. Mol. Pharm..

[9] Amendoeira A., García L.R., Fernandes A.R., Baptista P.V. (2020). Light Irradiation of Gold Nanoparticles Toward Advanced Cancer Therapeutics. Adv. Ther..

[10] Stolle H.L.K.S., Garwe F., Müller R., Krech T., Oberleiter B., Rainer T., Fritzsche W., Stolle A. (2018). Design of a scalable AuNP catalyst system for plasmon-driven photocatalysis. Rsc. Adv..

[11] Adleman J.R., Boyd D.A., Goodwin D.G., Psaltis D. (2009). Heterogenous Catalysis Mediated by Plasmon Heating. Nano Lett..

[12] Zhang Z., Zhang C., Zheng H., Xu H. (2019). Plasmon-Driven Catalysis on Molecules and Nanomaterials. Acc. Chem. Res..

[13] Christopher P., Xin H., Linic S. (2011). Visible-light-enhanced catalytic oxidation reactions on plasmonic silver nanostructures. Nat. Chem..

[14] Chen X., Cai Z., Chen X., Oyama M. (2014). AuPd bimetallic nanoparticles decorated on graphene nanosheets: Their green synthesis, growth mechanism and high catalytic ability in 4-nitrophenol reduction. J. Mater. Chem. A.

[15] Steinbrück A., Csáki A., Festag G., Fritzsche W. (2006). Preparation and Optical Characterization of Core-Shell Bimetal Nanoparticles. Plasmonics.

[16] Chen D.-H., Chen C.-J. (2002). Formation and characterization of Au–Ag bimetallic nanoparticles in water-in-oil microemulsions. J. Mater. Chem..

[17] Gonzalez C.M., Liu Y., Scaiano J.C. (2009). Photochemical Strategies for the Facile Synthesis of Gold−Silver Alloy and Core−Shell Bimetallic Nanoparticles. J. Phys. Chem. C.

[18] Rodríguez-González B., Burrows A., Watanabe M., Kiely C.J., Liz Marzán L.M. (2005). Multishell bimetallic AuAg nanoparticles: Synthesis, structure and optical properties. J. Mater. Chem..

[19] Wu M.-L., Chen D.-H., Huang T.-C. (2001). Synthesis of Au/Pd Bimetallic Nanoparticles in Reverse Micelles. Langmuir.

[20] Ferrer D., Torres-Castro A., Gao X., Sepúlveda-Guzmán S., Ortiz-Méndez U., José-Yacamán M. (2007). Three-Layer Core/Shell Structure in Au−Pd Bimetallic Nanoparticles. Nano Lett..

[21] Cao X., Wang N., Jia S., Guo L., Li K. (2013). Bimetallic AuPt nanochains: Synthesis and their application in electrochemical immunosensor for the detection of carcinoembryonic antigen. Biosens. Bioelectron..

[22] Santoveña-Uribe A., Maya-Cornejo J., Bahena D., Ledesma J., Pérez R., Esparza R. (2020). Synthesis and Characterization of AgPd Bimetallic Nanoparticles as Efficient Electrocatalysts for Oxygen Reduction Reaction. Electrocatalysis.

[23] Sivamaruthi B.S., Ramkumar V.S., Archunan G., Chaiyasut C., Suganthy N. (2019). Biogenic synthesis of silver palladium bimetallic nanoparticles from fruit extract of Terminalia chebula—In vitro evaluation of anticancer and antimicrobial activity. J. Drug Deliv. Sci. Technol..

[24] Yi Z., Xu X., Li X., Luo J., Wu W., Tang Y., Yi Y. (2011). Facile preparation of Au/Ag bimetallic hollow nanospheres and its application in surface-enhanced Raman scattering. Appl. Surf. Sci..

[25] Kang H., Jeong S., Park Y., Yim J., Jun B.-H., Kyeong S., Yang J.-K., Kim G., Hong S., Lee L.P. (2013). Near-Infrared SERS Nanoprobes with Plasmonic Au/Ag Hollow-Shell Assemblies for In Vivo Multiplex Detection. Adv. Funct. Mater..

[26] Fan M., Lai F.-J., Chou H.-L., Lu W.-T., Hwang B.-J., Brolo A.G. (2013). Surface-enhanced Raman scattering (SERS) from Au:Ag bimetallic nanoparticles: The effect of the molecular probe. Chem. Sci..

[27] Yan Y., Radu A.I., Rao W., Wang H., Chen G., Weber K., Wang D., Cialla-May D., Popp J., Schaaf P. (2016). Mesoscopically Bi-continuous Ag–Au Hybrid Nanosponges with Tunable Plasmon Resonances as Bottom-Up Substrates for Surface-Enhanced Raman Spectroscopy. Chem. Mater..

[28] Yu H., He Y. (2015). Seed-assisted synthesis of dendritic Au–Ag bimetallic nanoparticles with chemiluminescence activity and their application in glucose detection. Sens. Actuators B Chem..

[29] Steinbrück A., Stranik O., Csáki A., Fritzsche W. (2011). Sensoric potential of gold–silver core–shell nanoparticles. Anal. Bioanal. Chem..

[30] Sharma M., Pudasaini P.R., Ruiz-Zepeda F., Vinogradova E., Ayon A.A. (2014). Plasmonic Effects of Au/Ag Bimetallic Multispiked Nanoparticles for Photovoltaic Applications. Acs Appl. Mater. Interfaces.

[31] Nasrollahzadeh M., Azarian A., Maham M., Ehsani A. (2015). Synthesis of Au/Pd bimetallic nanoparticles and their application in the Suzuki coupling reaction. J. Ind. Eng. Chem..

[32] Zhang S., Metin Ö., Su D., Sun S. (2013). Monodisperse AgPd Alloy Nanoparticles and Their Superior Catalysis for the Dehydrogenation of Formic Acid. Angew. Chem. Int. Ed..

[33] Yang L., Hua X., Su J., Luo W., Chen S., Cheng G. (2015). Highly efficient hydrogen generation from formic acid-sodium formate over monodisperse AgPd nanoparticles at room temperature. Appl. Catal. B Environ..

[34] Wu D., Kusada K., Kitagawa H. (2016). Recent progress in the structure control of Pd–Ru bimetallic nanomaterials. Sci. Technol. Adv. Mater..

[35] González E., Arbiol J., Puntes V.F. (2011). Carving at the Nanoscale: Sequential Galvanic Exchange and Kirkendall Growth at Room Temperature. Science.

[36] Loza K., Heggen M., Epple M. (2020). Synthesis, Structure, Properties, and Applications of Bimetallic Nanoparticles of Noble Metals. Adv. Funct. Mater..

[37] Ghosh Chaudhuri R., Paria S. (2012). Core/Shell Nanoparticles: Classes, Properties, Synthesis Mechanisms, Characterization, and Applications. Chem. Rev..

[38] Rodríguez-González B., Sánchez-Iglesias A., Giersig M., Liz-Marzán L.M. (2004). AuAg bimetallic nanoparticles: Formation, silica-coating and selective etching. Faraday Discuss..

[39] Li G., Luo Y. (2008). Preparation and Characterization of Dendrimer-Templated Ag−Cu Bimetallic Nanoclusters. Inorg. Chem..

[40] Wang D., Villa A., Porta F., Prati L., Su D. (2008). Bimetallic Gold/Palladium Catalysts: Correlation between Nanostructure and Synergistic Effects. J. Phys. Chem. C.

[41] Liu P., Gu X., Zhang H., Cheng J., Song J., Su H. (2017). Visible-light-driven catalytic activity enhancement of Pd in AuPd nanoparticles for hydrogen evolution from formic acid at room temperature. Appl. Catal. B Environ..

[42] Zhu X., Guo Q., Sun Y., Chen S., Wang J.-Q., Wu M., Fu W., Tang Y., Duan X., Chen D. (2019). Optimising surface d charge of AuPd nanoalloy catalysts for enhanced catalytic activity. Nat. Commun..

[43] Csáki A., Berg S., Jahr N., Leiterer C., Schneider T., Steinbrück A., Zopf D., Fritzsche W., Chow P.E. (2010). Plasmonic Nanoparticles—Noble Material For Sensoring Applications. Gold Nanoparticles: Properties, Characterization and Fabrication.

[44] Daniel M.C., Astruc D. (2004). Gold nanoparticles: Assembly, supramolecular chemistry, quantum-size-related properties, and applications toward biology, catalysis, and nanotechnology. Chem. Rev..

[45] Yin L., Liebscher J. (2007). Carbon-carbon coupling reactions catalyzed by heterogeneous palladium catalysts. Chem. Rev..

[46] Balanta A., Godard C., Claver C. (2011). Pd nanoparticles for C–C coupling reactions. Chem. Soc. Rev..

[47] Kwak J.H., Kovarik L., Szanyi J. (2013). Heterogeneous Catalysis on Atomically Dispersed Supported Metals: CO_2_ Reduction on Multifunctional Pd Catalysts. Acs Catal..

[48] Erdoğan H., Metin Ö., Özkar S. (2009). In situ-generated PVP-stabilized palladium(0) nanocluster catalyst in hydrogen generation from the methanolysis of ammonia–borane. Phys. Chem. Chem. Phys..

[49] Nakamura T., Mochidzuki Y., Sato S. (2011). Fabrication of gold nanoparticles in intense optical field by femtosecond laser irradiation of aqueous solution. J. Mater. Res..

[50] Herbani Y., Nakamura T., Sato S. (2011). Synthesis of Near-Monodispersed Au–Ag Nanoalloys by High Intensity Laser Irradiation of Metal Ions in Hexane. J. Phys. Chem. C.

[51] McGilvray K.L., Decan M.R., Wang D., Scaiano J.C. (2006). Facile Photochemical Synthesis of Unprotected Aqueous Gold Nanoparticles. J. Am. Chem. Soc..

[52] Gellé A., Moores A. (2019). Plasmonic nanoparticles: Photocatalysts with a bright future. Curr. Opin. Green Sustain. Chem..

[53] Huang H.J., Wu J.C., Chiang H.-P., Chou Chau Y.-F., Lin Y.-S., Wang Y.H., Chen P.-J. (2020). Review of Experimental Setups for Plasmonic Photocatalytic Reactions. Catalysts.

[54] Mukherjee S., Libisch F., Large N., Neumann O., Brown L.V., Cheng J., Lassiter J.B., Carter E.A., Nordlander P., Halas N.J. (2013). Hot Electrons Do the Impossible: Plasmon-Induced Dissociation of H2 on Au. Nano Lett..

[55] Knauer A., Thete A., Li S., Romanus H., Csáki A., Fritzsche W., Köhler J.M. (2011). Au/Ag/Au double shell nanoparticles with narrow size distribution obtained by continuous micro segmented flow synthesis. Chem. Eng. J..

[56] Chen D., Li J., Cui P., Liu H., Yang J. (2016). Gold-catalyzed formation of core–shell gold–palladium nanoparticles with palladium shells up to three atomic layers. J. Mater. Chem. A.

[57] Henning A.M., Watt J., Miedziak P.J., Cheong S., Santonastaso M., Song M., Takeda Y., Kirkland A.I., Taylor S.H., Tilley R.D. (2013). Gold–Palladium Core–Shell Nanocrystals with Size and Shape Control Optimized for Catalytic Performance. Angew. Chem. Int. Ed..

[58] Gonzalez C.M., Martin B., Betancourt T. (2014). Photochemical synthesis of bimetallic and anisotropic Au-containing nanoparticles using a one-step protocol. J. Mater. Chem. A.

[59] Festag G., Steinbrück A., Csáki A., Möller R., Fritzsche W. (2006). Single particle studies of the autocatalytic metal deposition onto surface-bound gold nanoparticles reveal a linear growth. Nanotechnology.

[60] Dong Y., Yang X., Zhang Z., Li S. (2016). Photochemical Synthesis of Au@Pd Core-Shell Nanoparticles for Methanol Oxidation Reaction: The Promotional Effect of the Au Core. MATEC Web Conf..

[61] Ma T., Liang F., Chen R., Liu S., Zhang H. (2017). Synthesis of Au-Pd Bimetallic Nanoflowers for Catalytic Reduction of 4-Nitrophenol. Nanomaterials.

[62] Britto Hurtado R., Cortez-Valadez M., Flores-Lopez N.S., Flores-Acosta M. (2020). Agglomerates of Au-Pt bimetallic nanoparticles: Synthesis and antibacterial activity. Gold Bull..

[63] Bastús N.G., Comenge J., Puntes V.c. (2011). Kinetically Controlled Seeded Growth Synthesis of Citrate-Stabilized Gold Nanoparticles of up to 200 nm: Size Focusing versus Ostwald Ripening. Langmuir.

[64] Fang Y., Hoh J.H. (1998). Early Intermediates in Spermidine-Induced DNA Condensation on the Surface of Mica. J. Am. Chem. Soc..

[65] Hayat M.A. (1989). Colloidal Gold: Principles, Methods, and Applications.

[66] Gallagher P.K., Gross M.E. (1986). The thermal decomposition of palladium acetate. J. Therm. Anal..

[67] Golubev A.A., Khlebtsov B.N., Rodriguez R.D., Chen Y., Zahn D.R.T. (2018). Plasmonic Heating Plays a Dominant Role in the Plasmon-Induced Photocatalytic Reduction of 4-Nitrobenzenethiol. J. Phys. Chem. C.

