# Supplementary material for: Modification of Surface Bond Au Nanospheres by Chemically and Plasmonically Induced Pd Deposition
Source: Nanomaterials (Basel). 2021 Jan 18;11(1):245. doi: 10.3390/nano11010245 (PMC7831503; doi:10.3390/nano11010245)
Supplement: Supplementary file 1 [file nanomaterials-11-00245-s001.pdf]

Supplementary Materials

Modification of Surface Bond Au Nanospheres by Chemically and Plasmonically Induced Pd Deposition

Heike Lisa Kerstin Stephanie Stolle [1], Andrea Csáki [1], Jan Dellith [2] and Wolfgang Fritzsche [1,*]

[1] Department of Nanobiophotonics, Leibniz Institute of Photonic Technology (IPHT), Albert-Einstein-Straße 9, D-07745 Jena, Germany; lisa.stolle@leibniz-ipht.de (H.L.K.S.S.); andrea.csaki@leibniz-ipht.de (A.C.)

[2] Competence Center for Micro- and Nanotechnologies, Leibniz Institute of Photonic Technology (IPHT), Albert-Einstein-Straße 9, D-07745 Jena, Germany; jan.dellith@leibniz-ipht,de

* Correspondence: wolfgang.fritzsche@leibniz-ipht.de; Tel.: +49-(0)3641-206-304

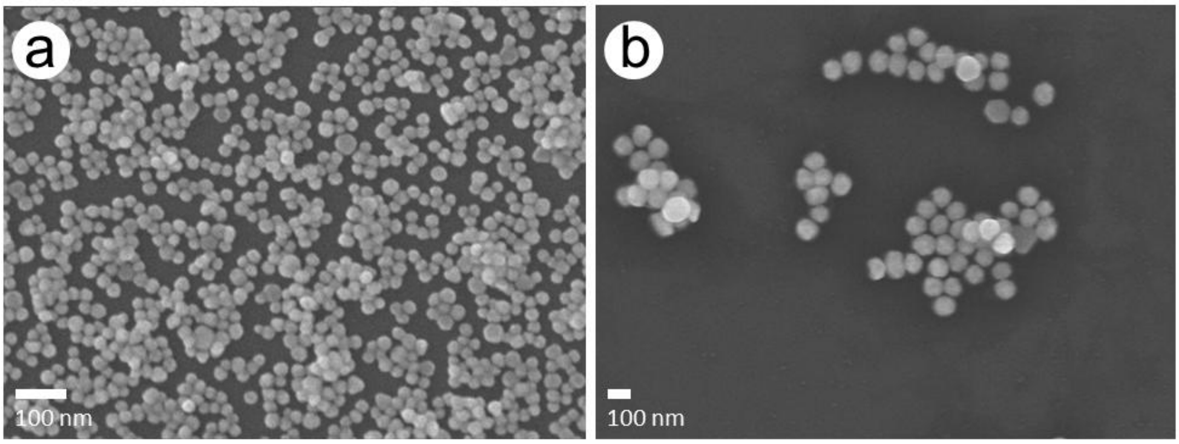

Figure S1. SEM images of utilized gold nanospheres (a) 30 nm and (b) 80 nm.

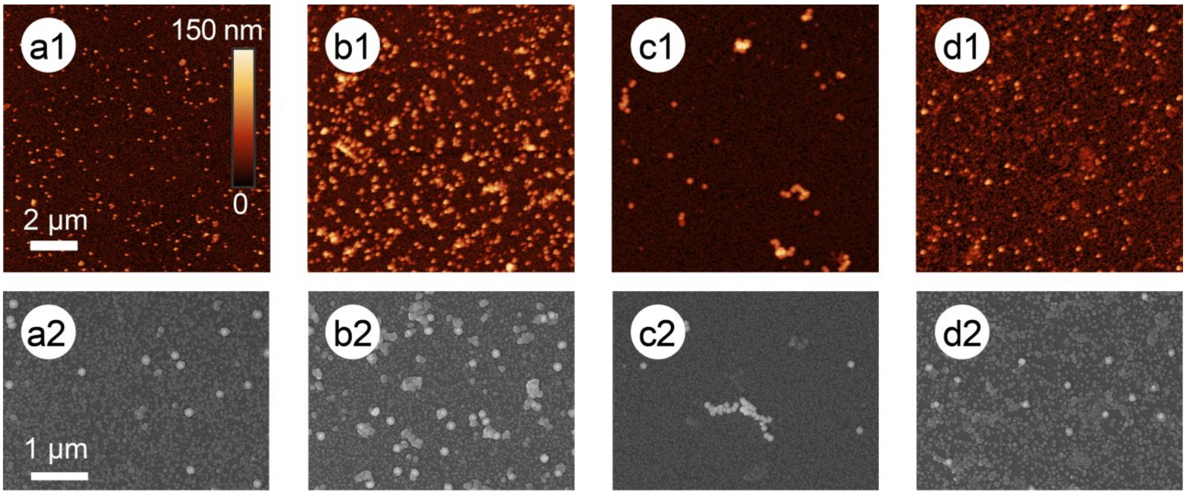

Figure S2. AFM images (a1-d1) and SEM images (a2-d2) of 80 nm AuNPs after ten minutes of enhancement in 2 min steps. Images a: 57 mM AS and 2.5 mM Pd acetate, images b: 114 mM AS and 2.5 mM PdAc, images c: 228 mM AS and 2.5 Pd acetate, images d: 228 mM and 1.0 mM Pd acetate. The scale bar of image S 2 a1 pertains to all four images.

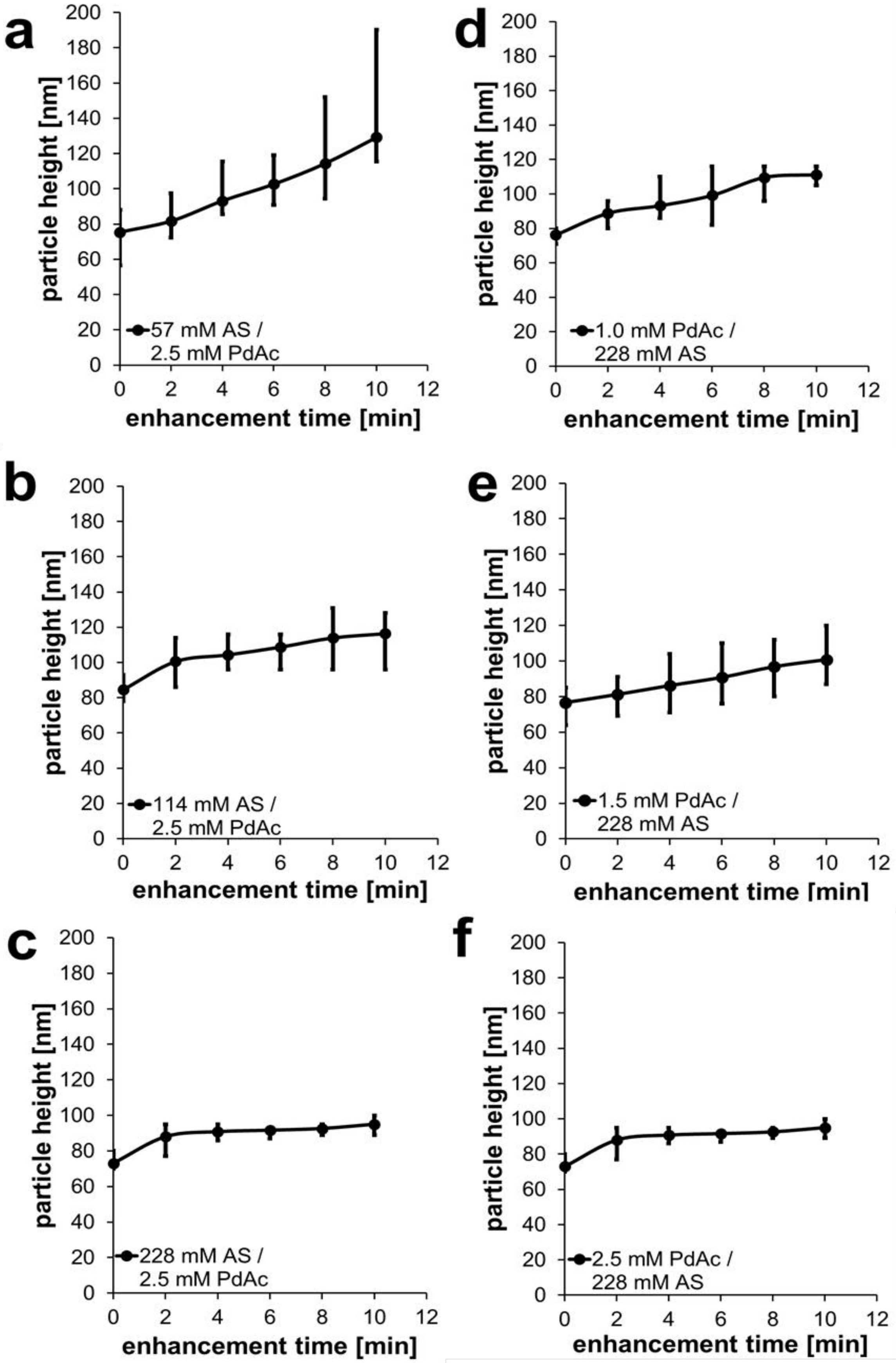

Figure S3. Effect of reducing agent concentration on particle height development (**a**-**c**) at constant (2.5 mM) Pd acetate concentration and (**d**-**f**) at constant ascorbic acid concentration of 228 mM.

Table S1. Total particle height, height of Pd shell and growth kinetics [nm/min] of 2 min enhancement steps up to 10 min for Pd enhancement with ascorbic acid.

enhancement time [min] in 2min steps	average particle height [nm]	average height of Pd shell [nm]	shell growth rate per 2 min step [nm/min]	shell growth rate over complete reaction time [nm/min]
**57 mM AS / 2.5 mM PdAc**				
**0**	75.3	0	0	0
**2**	81.6	6.30	3.15	3.15
**4**	93.2	17.9	5.80	4.48
**6**	102.6	27.3	4.70	4.55
**8**	114.4	39.1	5.90	4.89
**10**	129.3	54	7.45	5.40
**114 mM AS / 2.5 mM PdAc**				
**0**	84.6	0	0	0
**2**	100.6	16.0	8.0	8.0
**4**	104.4	19.8	1.90	4.95
**6**	108.8	24.2	2.20	4.03
**8**	114.0	29.4	2.60	3.68
**10**	116.4	31.8	1.20	3.18
**228 mM AS / 2.5 mM PdAc**				
**0**	73.0	0	0	0
**2**	88.0	15.0	7.50	7.50
**4**	90.8	17.8	1.40	4.45
**6**	91.6	18.6	0.40	3.10
**8**	92.6	19.6	0.50	2.45
**10**	95.0	22.0	1.20	2.20
**1.5 mM PdAc / 228 mM AS**				
**0**	76.6	0	0	0
**2**	81.2	4.60	2.30	2.30
**4**	86.2	9.60	2.50	2.40
**6**	90.8	14.2	2.30	2.37
**8**	96.8	20.2	3.00	2.53
**10**	100.8	24.2	2.00	2.42
**1.0 mM PdAc / 228 mM AS**				
**0**	76.2	0	0	0
**2**	88.8	12.6	6.30	6.30
**4**	93.2	17.0	2.20	4.25
**6**	99.2	23.0	3.00	3.83
**8**	109.6	33.4	5.20	4.18
**10**	111.2	35.0	0.80	3.50

Table S2. Total particle height, height of Pd shell and growth kinetics [nm/min] of 2 min enhancement steps up to 10 min of 80 nm AuNPs under white light and of 30 nm AuNPs under green light.

enhancement time [min] in 2min steps	average particle height [nm]	average height of Pd shell [nm]	shell growth rate per 2 min step [nm/min]	shell growth rate over complete reaction time [nm/min]
**white light / 80 nm AuNPs**				
**0**	77.1	0	0	0
**2**	79.2	2.10	1.05	1.05
**4**	81.3	4.20	1.05	1.05
**6**	82.6	5.50	0.65	0.92
**8**	85.5	8.40	0.76	1.05
**10**	88.7	11.6	1.60	1.16
**green light / 30 nm AuNPs**				
**0**	23.2	0	0	0
**2**	24.1	0.90	0.45	0.45
**4**	24.9	1.70	0.40	0.43
**6**	25.7	2.50	0.40	0.42
**8**	25.9	2.70	0.40	0.34
**10**	26.4	3.20	0.25	0.32

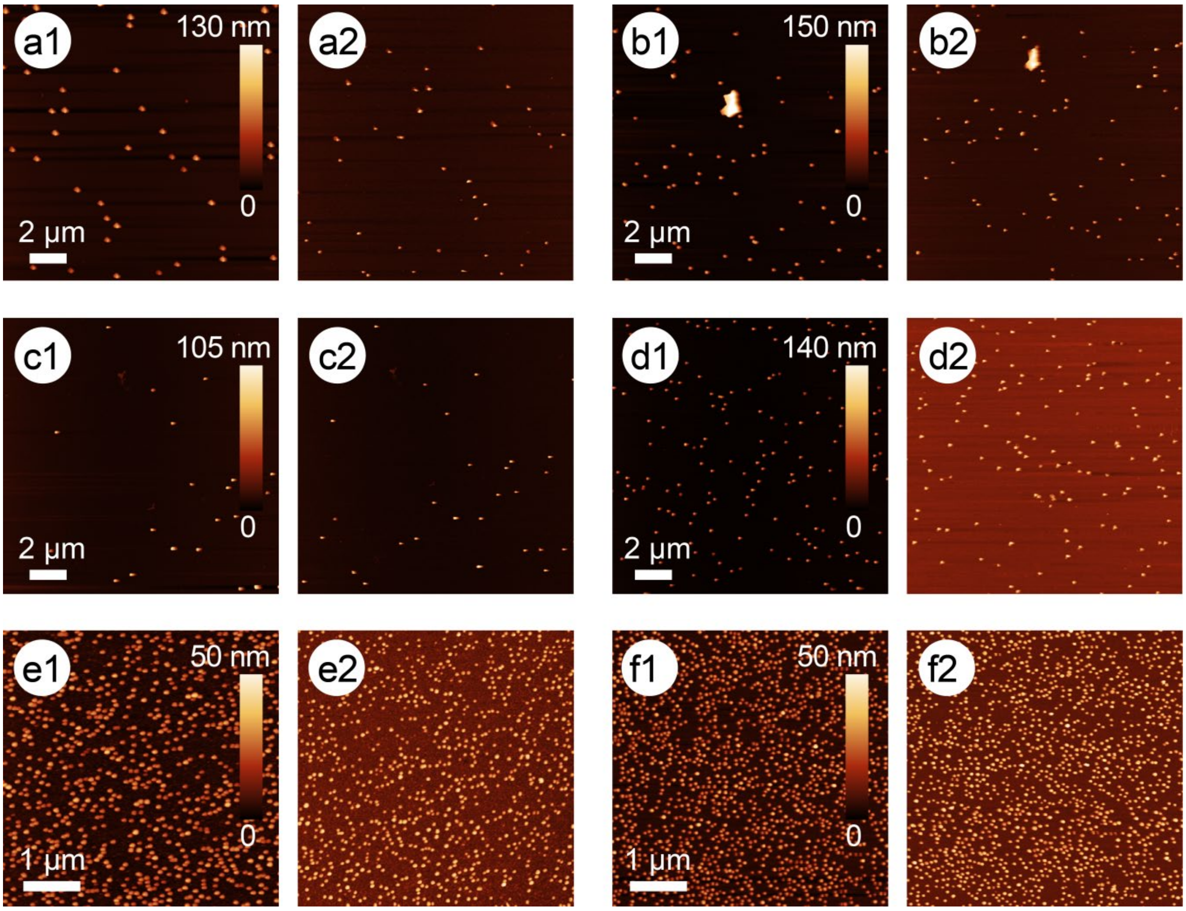

Figure S4. AFM images of Pd enhancement with light at 0 and 10 min (without 2 min steps) reaction time (a1-2: 80 nm AuNPs / white light, b1-2: 80 nm AuNPs / blue LEDs, c1-2: 80 nm AuNPs / red LEDs, d1-2: 80 nm AuNPs / green LEDs, e1-2: 30 nm AuNPs / green LEDs, f1-2: 30 nm AuNPs / white light). The scale bar displayed in the respective first image is analogous within each set of images.

Evaluation of temperature effects

The temperatures of both the chrome grid chips and the Pd acetate solution were measured with an infrared thermometer. Table S3 lists the measured temperatures before and after irradiation.

Table S3. Temperature values [°C] and differences [K] of the chrome grid chip and the Pd acetate solution before and after irradiation during experiments.

	T_0 [°C] PdAc solution / chip	T_x [°C] PdAc solution / chip	ΔT [K] PdAc solution / chip
**2 min white light**	20.1 / 20.4	20.2 / 20.4	0.1 / 0
**10 min white light**	20.1 / 20.4	20.5 / 20.4	0.4 / 0
**2 min green light**	20.3 / 20.3	20.3 / 20.3	0 / 0
**10 min green light**	20.3 / 20.3	23.0 / 23.0	2.7 / 2.7
**10 min blue light**	21.0 / 21.0	25.2 / 22.8	4.2 / 1.7
**10 min red light**	20.5 / 20.5	22.9 / 22.0	2.4 / 1.5

As it can be seen, the resulting temperature differences are all beyond 5 K, which speaks against a considerable effect through plasmonic heating or through the utilized light sources. The highest measured was 4.2 K for the PdAc solution for the blue LEDs, followed by 2.7 K for green LEDs and 2.4 K for red LEDs for 10 min irradiation time each. The temperature differences measured in case of the white light are overall negligible and even zero at an irradiation time of 2 min with green LEDs. The temperature differences measured on the chip surface are even lower compared to the solution. Regarding the results for white light and an irradiation time of two minutes with green LEDs, there could not be measured any temperature difference. For ten minutes enhancement time with green, blue and red LEDs, a temperature difference of 2.7, 1.7 and 1.5 K was measured respectively. The differences between chip and solution temperature can be explained by the specific heat capacity of the different media, which is with 4.2 kJ/kg·K for water[1] (neglecting the ions inside) higher than for the chip consisting of silicon (0.7 kJ/ kg·K)[2]. But also the absorption of the PdAc solution plays a role. Regarding the UV/Vis spectrum (Figure S5), it can be seen that the solution is mostly absorbing at lower wavelengths (200 – 300 nm) but also up to a wavelength of 700 nm. Therefore, the radiation of each of the utilized lamps can contribute to a heating of the PdAc solution, especially visible for the blue LED array as most powerful array, but also for the rather weak green LEDs. In case of the red LEDs, the absorption at the emitted wavelength of 628 nm is lower and therefore, the temperature difference less.

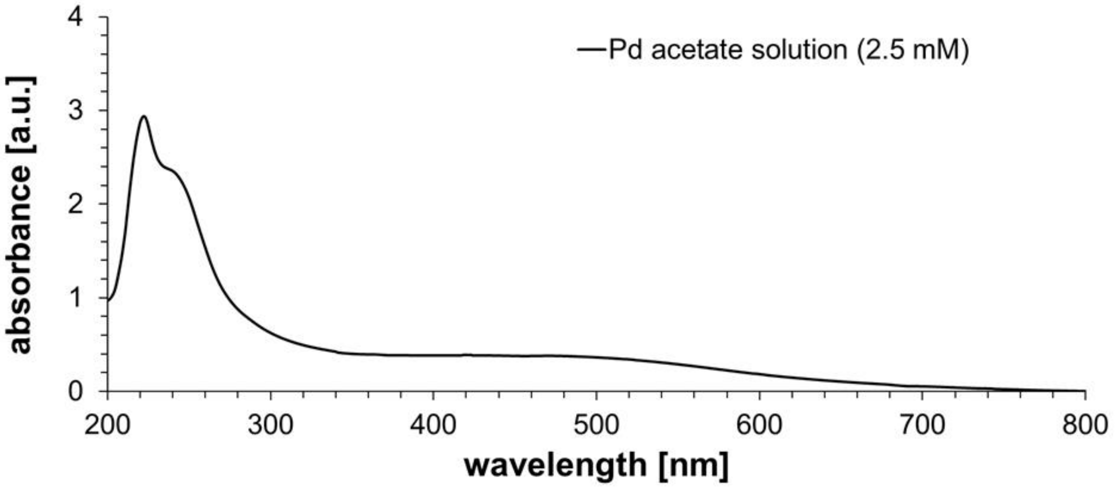

Figure S5. UV/Vis spectrum of Pd acetate solution (2.5 mM).

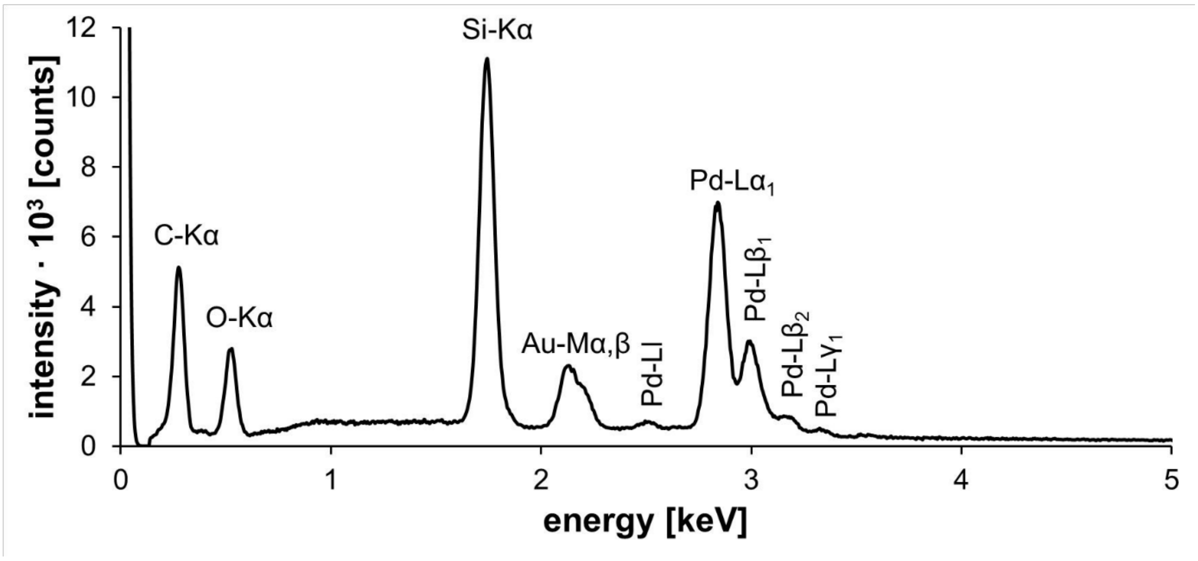

Figure S6. EDX spectrum of a nanoparticle cluster on the chip with 80 nm AuNPs and 114 mM AS / 2.5 mM PdAc (carbon signal due to carbon coating of the sample, oxygen signal due to oxidized silicon and silicon signal itself from the chip).

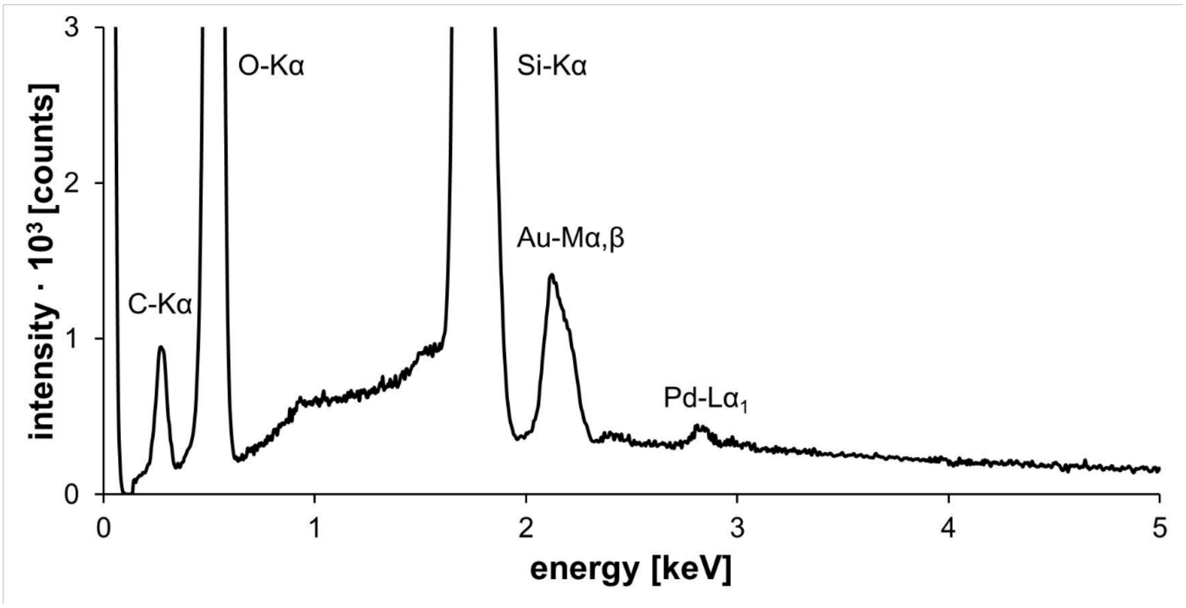

Figure S7. EDX spectrum of a chosen nanoparticle on the chip with 80 nm AuNPs and 2.5 mM PdAc and step-wise irradiation with white light (carbon signal due to carbon coating of the sample, oxygen signal due to oxidized silicon and silicon signal itself from the chip). .

References

1. Cornillon, P.; Andrieu, J.; Duplan, J.C.; Laurent, M. Use of nuclear magnetic resonance to model thermophysical properties of frozen and unfrozen model food gels. *Journal of Food Engineering* **1995**, *25*, 1-19, doi:10.1016/0260-8774(94)00004-S.
2. Shin, D.; Banerjee, D. Enhanced Specific Heat Capacity of Nanomaterials Synthesized by Dispersing Silica Nanoparticles in Eutectic Mixtures. *Journal of Heat Transfer* **2013**, *135*, doi:10.1115/1.4005163.
